# Anesthetic Propofol Attenuates the Isoflurane-Induced Caspase-3 Activation and Aβ Oligomerization

**DOI:** 10.1371/journal.pone.0027019

**Published:** 2011-11-01

**Authors:** Yiying Zhang, Yu Zhen, Yuanlin Dong, Zhipeng Xu, Yun Yue, Todd E. Golde, Rudolph E. Tanzi, Robert D. Moir, Zhongcong Xie

**Affiliations:** 1 Geriatric Anesthesia Research Unit, Department of Anesthesia, Critical Care and Pain Medicine, Massachusetts General Hospital and Harvard Medical School, Charlestown, Massachusetts, United States of America; 2 Genetics and Aging Research Unit, MassGeneral Institute for Neurodegenerative Disease, Department of Neurology, Massachusetts General Hospital and Harvard Medical School, Charlestown, Massachusetts, United States of America; 3 Department of Anesthesia, Beijing Friendship Hospital, Capital Medical University, Beijing, People's Republic of China; 4 Department of Anesthesia, Beijing Chaoyang Hospital, Capital Medical University, Beijing, People's Republic of China; 5 Center for Translational Research in Neurodegenerative Disease, Department of Neuroscience, University of Florida, Gainesville, Florida, United States of America; University of Nebraska Medical Center, United States of America

## Abstract

Accumulation and deposition of β-amyloid protein (Aβ) are the hallmark features of Alzheimer's disease. The inhalation anesthetic isoflurane has been shown to induce caspase activation and increase Aβ accumulation. In addition, recent studies suggest that isoflurane may directly promote the formation of cytotoxic soluble Aβ oligomers, which are thought to be the key pathological species in AD. In contrast, propofol, the most commonly used intravenous anesthetic, has been reported to have neuroprotective effects. We therefore set out to compare the effects of isoflurane and propofol alone and in combination on caspase-3 activation and Aβ oligomerization *in vitro* and *in vivo*. Naïve and stably-transfected H4 human neuroglioma cells that express human amyloid precursor protein, the precursor for Aβ; neonatal mice; and conditioned cell culture media containing secreted human Aβ40 or Aβ42 were treated with isoflurane and/or propofol. Here we show for the first time that propofol can attenuate isoflurane-induced caspase-3 activation in cultured cells and in the brain tissues of neonatal mice. Furthermore, propofol-mediated caspase inhibition occurred when there were elevated levels of Aβ. Finally, isoflurane alone induces Aβ42, but not Aβ40, oligomerization, and propofol can inhibit the isoflurane-mediated oligomerization of Aβ42. These data suggest that propofol may mitigate the caspase-3 activation by attenuating the isoflurane-induced Aβ42 oligomerization. Our findings provide novel insights into the possible mechanisms of isoflurane-induced neurotoxicity that may aid in the development of strategies to minimize potential adverse effects associated with the administration of anesthetics to patients.

## Introduction

Alzheimer's disease (AD) is one of the most common forms of dementia, which affects 4.5 million Americans and costs more than $100 billion a year on direct patient care alone. Accumulation and oligomerization of β-amyloid protein (Aβ), tau protein hyperphosphorylation, neuronal loss, synaptic dysfunction, mitochondrial damage, neuroinflammation, loss of calcium regulation, and other changes have been shown to contribute to AD neuropathogenesis (reviewed in [1]). Increasing evidence suggests a role for caspase activation and apoptosis in AD neuropathogenesis ([2], [3], [4], [5], [6], [7], [8], [9], [10], [11], reviewed in [12], [13]). A recent study by Burguillos et al. [14] have shown that pro-inflammatory stimuli, e.g., lipopolysaccharide, can induce the activation of caspase-8, -3 and -7 inside microglia without causing apoptosis. The activated caspases then induce microglia activation via the protein kinase C-d-dependent pathway. In addition, the activated caspases have been found in the frontal cortex of AD patients. Taken together, these findings suggest that caspase activation alone without apoptosis may still be able to contribute to AD neuropathogenesis.

Several studies have shown that the commonly used inhalation anesthetic isoflurane may induce caspase activation, apoptosis, Aβ oligomerization and accumulation, neuroinflammation, tau protein hyperphosphorylation, mitochondrial dysfunction, and impairment of learning and memory ([15], [16], [17], [18], [19], [20], [21], [22], [23], reviewed in [24], [25]). However, the underlying mechanisms and potential interventions of these effects remain largely to be determined.

Propofol, the most commonly used intravenous anesthetics, has been shown to have neuroprotective effects by attenuating caspase activation and apoptosis [26], [27], [28], [29], [30], [31], [32]. However, the effects of propofol on the isoflurane-induced caspase activation, as well as the potential underlying mechanisms, have not yet been investigated.

In the present studies, we set out to determine the effects of propofol on the isoflurane-induced caspase-3 activation in human neuroglioma cells and in the brain tissues of neonatal mice. Moreover, we performed mechanistic studies to determine whether propofol can attenuate the isoflurane-induced caspase-3 activation by affecting isoflurane's effects on Aβ oligomerization.

## Results

### Propofol attenuates the isoflurane-induced caspase-3 activation in H4-APP cells

Several studies have shown that isoflurane can induce caspase activation [18], [19], [33], [34], [35], [36], [37], [38]. The molecular mechanisms underlying these effects, however, are largely unknown. Moreover, interventions that could mitigate the isoflurane-induced caspase-3 activation remain largely to be determined. Propofol has been shown to have neuroprotective effects by attenuating caspase activation and apoptosis [26], [27], [28], [29], [30], [31], [32]. We therefore assessed whether propofol can mitigate the isoflurane-induced caspase-3 activation *in vitro* (H4-APP and H4 naïve cells) and *in vivo* [neonatal wild-type (WT) and AD transgenic (Tg) mice].

**Figure 1:**
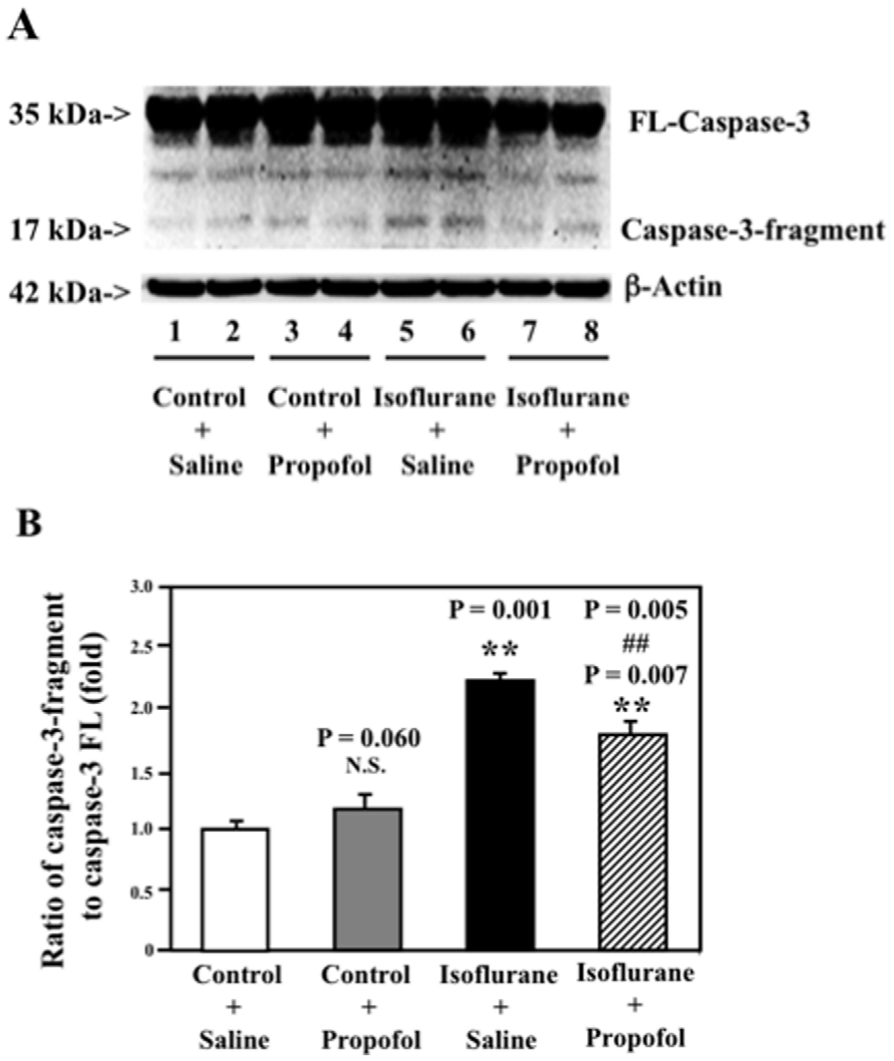
Propofol attenuates the isoflurane-induced caspase-3 activation in H4-APP cells. *A.* Treatment with 2% isoflurane (lanes 5 and 6) induces caspase-3 activation as compared to the control condition (lanes 1 and 2). The treatment of propofol alone (lanes 3 and 4) does not induce caspase-3 activation as compared to the control condition (lanes 1 and 2). However, propofol treatment (lanes 7 and 8) attenuates the 2% isoflurane-induced caspase-3 activation as compared to 2% isoflurane alone (lanes 5 and 6) in the H4-APP cells. There is no significant difference in amounts of β-Actin in the H4-APP cells following these treatments. *B.* Quantification of the Western blot shows that 2% isoflurane (black bar, ** P = 0.001) induces caspase-3 activation as compared to control condition (white bar). The propofol treatment alone (gray bar) does not induce caspase-3 activation as compared to control condition (white bar). However, two-way ANOVA shows that the propofol treatment (net bar, ## P = 0.005) attenuates the isoflurane-induced caspase-3 activation. (N = 3).

The H4-APP cells were treated with 100 nM propofol or saline for 10 minutes following by 2% isoflurane or control condition for six hours. The cells were harvested at the end of the experiment and were subjected to Western blot analysis. Caspase-3 immunoblotting revealed that the isoflurane treatment induced caspase-3 activation (Figure 1A) as evidenced by increased ratios of cleaved (activated) caspase-3 fragment (17 kDa) to full-length (FL) (35–40 kDa) caspase-3. Treatment with 100 nM propofol alone did not induce caspase-3 activation, but the propofol treatment attenuated the isoflurane-induced caspase-3 activation (Figure 1A). Quantification of the Western blots (Figure 1B), based on the ratio of caspase-3 fragment to FL caspase-3, revealed that isoflurane (black bar) led to caspase-3 activation as compared to the control condition (white bar): 1.00 versus 2.30 fold (P = 0.001). The propofol treatment (net bar) attenuated the isoflurane-induced caspase-3 activation: 2.30 fold versus 1.77 fold (P = 0.005). These findings suggest that propofol may mitigate the isoflurane-induced caspase-3 activation in H4-APP cells.

### Propofol does not attenuate the isoflurane-induced caspase-3 activation in H4 naïve cells

**Figure 2:**
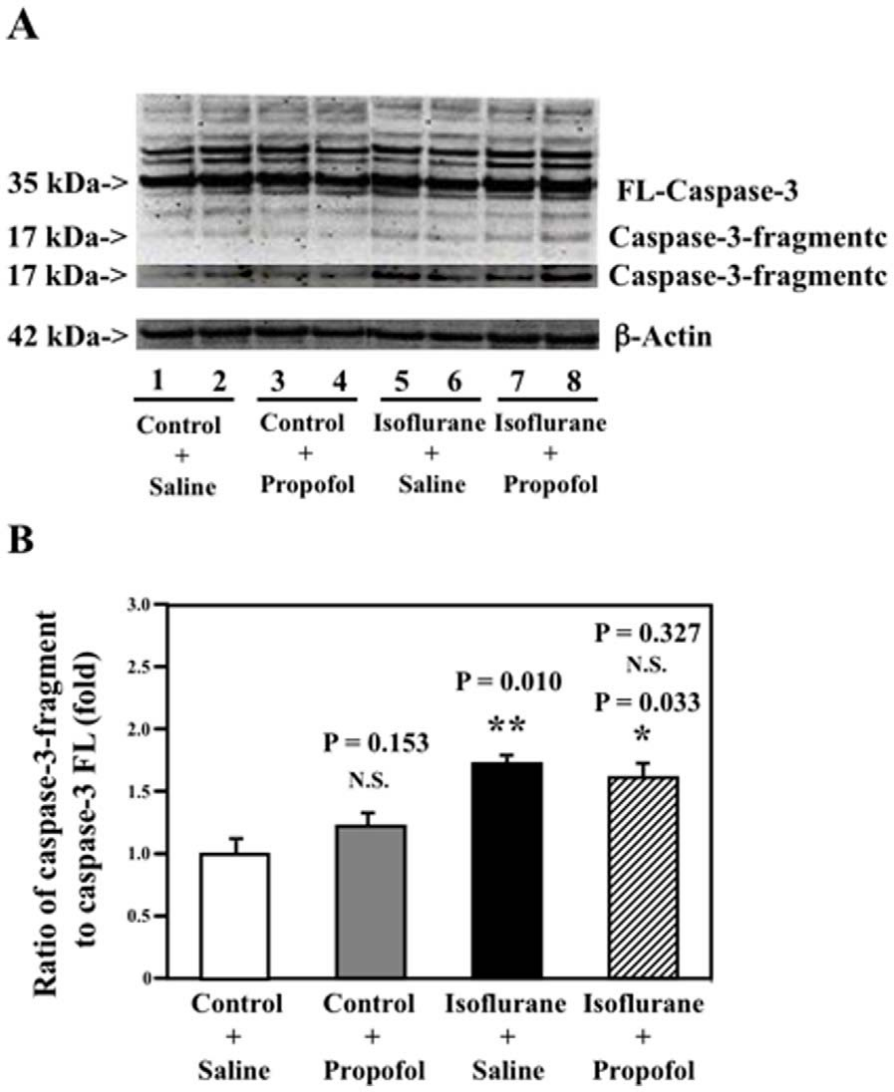
Propofol does not attenuate the isoflurane induced caspase-3 activation in H4 naive cells. *A.* Treatment with 2% isoflurane (lanes 5 and 6) induces caspase-3 activation as compared to the control condition (lanes 1 and 2). The treatment of propofol alone (lanes 3 and 4) does not induce caspase-3 activation as compared to the control condition (lanes 1 and 2). Propofol treatment (lanes 7 and 8) does not attenuate the 2% isoflurane-induced caspase-3 activation as compared to 2% isoflurane alone (lanes 5 and 6) in the H4 naive cells. There is no significant difference in amounts of β-Actin in the H4 naive cells following these treatments. *B.* Quantification of the Western blot shows that 2% isoflurane (black bar, ** P = 0.010) induces caspase-3 activation as compared to control condition (white bar). The propofol treatment alone (gray bar) does not induce caspase-3 activation as compared to the control condition (white bar). Two-way ANOVA shows that the propofol treatment (net bar, P = 0.327, N.S.) does not attenuate the isoflurane-induced caspase-3 activation in the H4 naïve cells. (N = 3).

Next, we assessed whether propofol could also attenuate the isoflurane-induced caspase-3 activation in H4 naïve cells, which have less Aβ levels than the H4-APP cells. Caspase-3 immunoblotting revealed that the isoflurane treatment induced caspase-3 activation as compared to the control condition in the H4 naïve cells (Figure 2A), which is consistent with our previous studies [37]. However, the propofol treatment did not attenuate the isoflurane-induced caspase-3 activation in the H4 naïve cells (Figure 2A). Quantification of the Western blot showed that isoflurane (black bar) led to caspase-3 activation as compared to the control condition (white bar): 1.00 versus 1.68 fold (P = 0.010). The propofol treatment (net bar) did not attenuate the isoflurane-induced caspase-3 activation: 1.68 versus 1.56 fold. These results showed that propofol did not mitigate the isoflurane-induced caspase-3 activation in H4 naive cells. Taken together, these findings suggest that propofol may have different effects in affecting the isoflurane-induced caspase-3 activation between the H4-APP and H4 naïve cells.

### The comparison of the effects of propofol between H4 naïve and H4-APP cells

Given the observation that propofol may have different effects when interacting with isoflurane between H4-APP cells and H4 naïve cells, we next performed studies to assess the effects of propofol on isoflurane-induced caspase-3 activation in both H4-APP and H4 naïve cells at the same time and compare their effects side by side in the Western blot analysis. We were able to show that propofol attenuated the isoflurane-induced caspase-3 activation in H4-APP cells, but not in H4 naïve cells (Figure S1A and S1B).

### The comparison of the effects of propofol between WT neonatal mice and AD Tg neonatal mice

**Figure 3:**
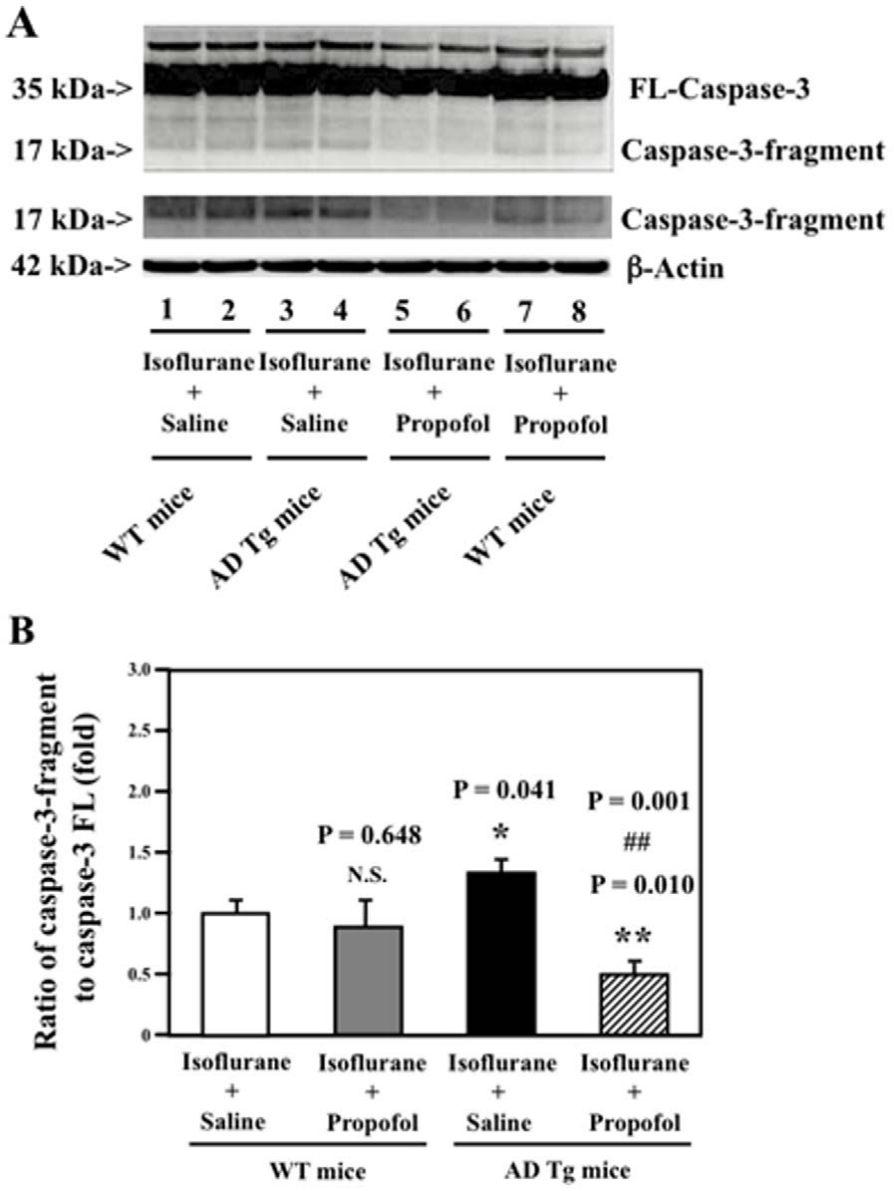
Propofol attenuates the isoflurane-induced caspase-3 activation in AD Tg neonatal mice but not in WT neonatal mice. *A.* In WT neonatal mice, the propofol treatment (lanes 7 and 8) does not attenuate the isoflurane-induced caspase-3 activation (lanes 1 and 2). Isoflurane induces a greater degree of caspase-3 activation in the AD Tg neonatal mice (lanes 3 and 4) as compared to that in WT neonatal mice (lanes 1 and 2). The propofol treatment (lanes 5 and 6) attenuates the isoflurane-induced caspase-3 activation (lanes 3 and 4) in the AD Tg neonatal mice. *B.* Quantification of the Western blot shows that propofol (grey bar) does not attenuate the isoflurane-induced caspase-3 activation (white bar) in the WT neonatal mice. Isoflurane induces a greater degree of caspase-3 activation in the AD Tg neonatal mice (black bar, * P = 0.041) than in the WT neonatal mice (white bar). In the AD Tg neonatal mice, propofol (net bar, ## P = 0.001) attenuates the isoflurane-induced caspase-3 activation (black bar). (N = 3).

Next, we performed the *in vivo* relevance studies by assessing the effects of isoflurane and propofol on caspase-3 activation in WT neonatal mice and AD Tg neonatal mice. We chose the neonatal mice in these experiments because the neonatal AD Tg mice are less expensive and are easier to obtain than the adult AD Tg mice. As can be seen in Figure 3A, propofol (lanes 5 and 6) attenuated the isoflurane-induced caspase-3 activation (lanes 3 and 4) in the brain tissues of AD Tg neonatal mice. However, the propofol treatment (lanes 7 and 8) did not attenuate the isoflurane-induced caspase-3 activation (lanes 1 and 2) in the brain tissues of WT neonatal mice. Quantification of the Western blot further illustrated that the propofol treatment (net bar) attenuated the isoflurane-induced caspase-3 activation in the AD Tg neonatal mice (black bar): 1.48 versus 0.5 fold, P = 0.001 (Figure 3B). In WT neonatal mice, however, the propofol treatment (gray bar) did not attenuate the isoflurane-induced caspase-3 activation: 1.00 versus 0.88 fold (Figure 3B). These results from the *in vivo* studies further suggest that elevated levels of Aβ may potentiate the isoflurane-induced caspase-3 activation, and the effects of propofol on attenuating the isoflurane-induced caspase-3 activation are dependent on the elevated levels of Aβ.

In order to rule out the possibility that isoflurane itself might not be activating caspase-3 above basal levels in WT and AD Tg mice, we assessed the effects of isoflurane alone on caspase-3 activation in the mice and we were able to show that anesthesia with 1.4% isoflurane for six hours induced caspase-3 activation in both WT and AD Tg mice (Figure S2A, S2B, S2C and S2D).

### Propofol attenuates the isoflurane-induced Aβ42 oligomerization

Isoflurane has been shown to enhance Aβ oligomerization in cultured cells [17]. iAβ5, clioquinol, and congo red, the inhibitors of Aβ oligomerization and aggregation, have been shown to mitigate the isoflurane-induced caspase-3 activation in cultured cells [36], [37]. Given the observation that propofol can attenuate the isoflurane-induced caspase-3 activation in the current experiment, next we asked whether propofol can reduce the isoflurane-induced Aβ oligomerization. The cell culture media containing secreted human Aβ40 or Aβ42, which resulted from the H4 cell overexpressed human Aβ40 or Aβ42, were treated with isoflurane and/or propofol for six hours. The media were harvested at the end of the experiments.

**Figure 4:**
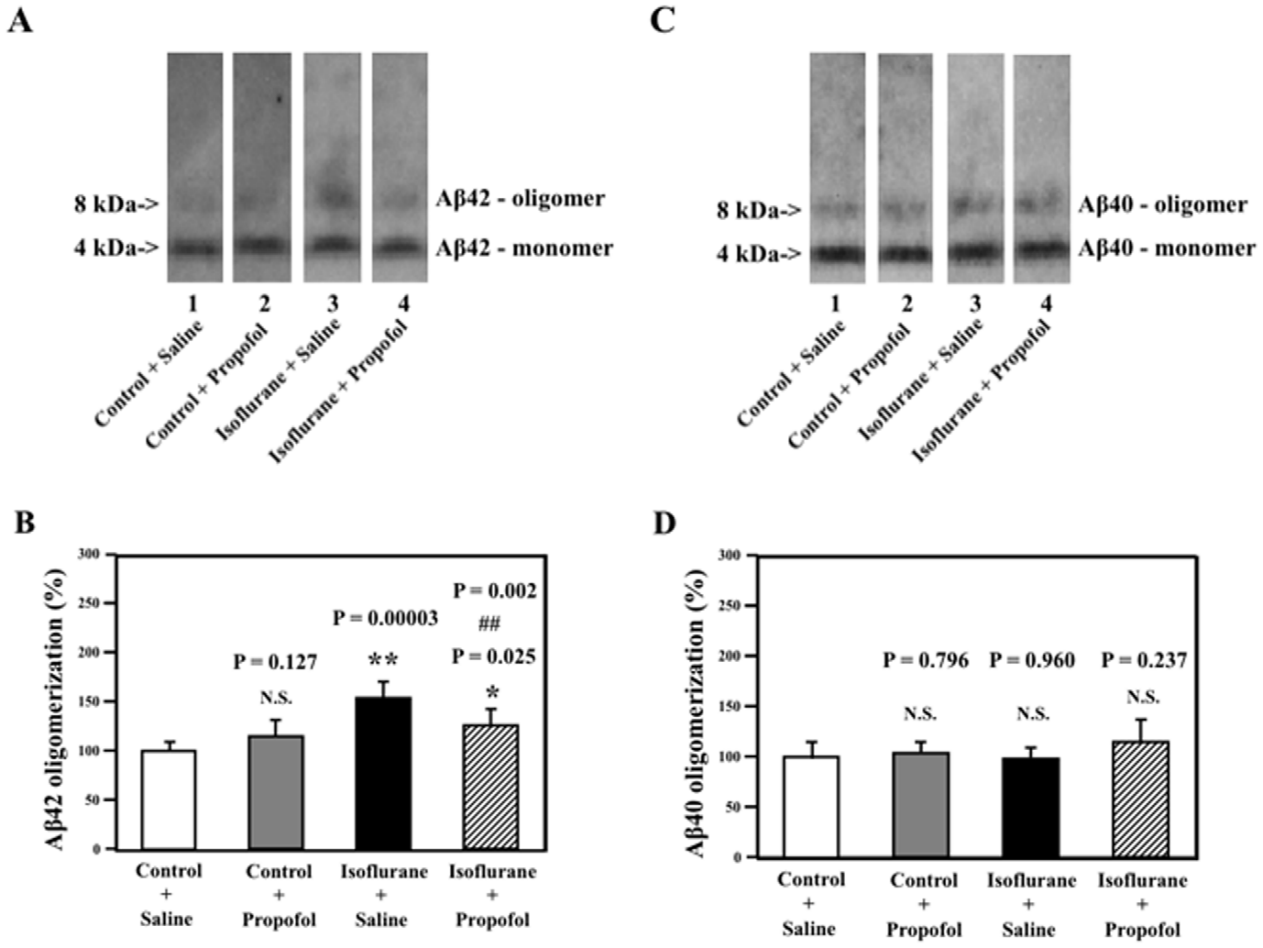
Propofol attenuates the isoflurane-induced Aβ42, but not Aβ40, oligomerization, in conditioned cell culture media. *A.* Treatment with 2% isoflurane for six hours (lanes 3) induces Aβ42 oligomerization as compared to the control condition (lanes 1). The treatment of propofol alone (lanes 2) does not affect Aβ42 oligomerization as compared to the control condition (lanes 1). However, the propofol treatment (lanes 4) attenuates the 2% isoflurane-induced Aβ42 oligomerization as compared to 2% isoflurane alone (lanes 3). *B.* Quantification of the Western blot shows that 2% isoflurane (black bar) induces Aβ42 oligomerization as compared to control condition (white bar), ** P = 0.00003. The propofol treatment alone (gray bar) does not affect Aβ42 oligomerization as compared to control condition (white bar). However, two-way ANOVA shows that the propofol treatment (net bar) attenuates the isoflurane-induced Aβ42 oligomerization, # P = 0.002. *C.* Treatment with 2% isoflurane alone (lanes 3), propofol alone (lane 2) or isoflurane plus propofol (lane 4) do not affect Aβ40 oligomerization as compared to the control condition (lanes 1). *D.* Quantification of the Western blot 2shows that 2% isoflurane alone (black bar), propofol alone (gray bar) or propofol plus isoflurane (net bar) do not affect Aβ40 oligomerization as compared to control condition (white bar). (N = 6).

As can be seen in Figure 4A, Aβ immunoblotting revealed that the isoflurane treatment increased Aβ42 oligomerization (lane 3) as compared to the control condition (lane 1). Treatment with 100 nM propofol alone (lane 2) did not affect Aβ42 oligomerization, but the propofol treatment (lane 4) attenuated the isoflurane-induced Aβ42 oligomerization. Quantification of the Western blots (Figure 4B) showed that the isoflurane treatment (black bar) led to an enhancement of Aβ42 oligomerization as compared to the control condition (white bar): 100% versus 150% (P = 0.00003). The propofol treatment (net bar) attenuated the isoflurane-induced Aβ42 oligomerization: 150% versus 128% (P = 0.025). These findings suggest that propofol may mitigate the isoflurane-induced Aβ42 oligomerization.

In contrast, Aβ immunoblotting revealed that the isoflurane treatment (Figure 4C, lane 3) did not enhance the Aβ40 oligomerization as compared to the control condition (Figure 4C, lane 1). Neither the propofol treatment alone (lane 2) nor the treatment of propofol plus isoflurane (lane 4) affected Aβ40 oligomerization as compared to control condition (lane 1) (Figure 4C). Quantification of the Western blots (Figure 4D) showed that the isoflurane treatment (black bar) did not enhance Aβ40 oligomerization as compared to the control condition (white bar). The treatments with propofol alone (gray bar) or propofol plus isoflurane (net bar) did not affect the Aβ40 oligomerization.

Finally, the concentration of Aβ40 (4.6±0.69 ng/ml) was not significantly different from that of Aβ42 (5.3±0.42 ng/ml), P = 0.090, N.S., in the experiments. Taken together, these findings suggest that isoflurane may specifically enhance Aβ42, but not Aβ40, oligomerization. Propofol can attenuate the isoflurane-induced Aβ42 oligomerization.

**Figure 5:**
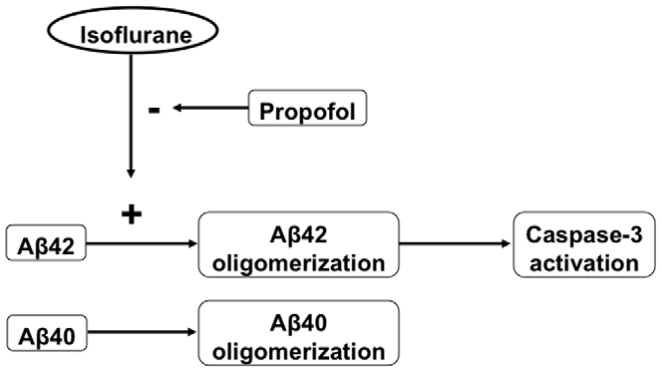
Hypothetical pathway by which propofol attenuates the isoflurane-induced caspase-3 activation. Isoflurane can induce oligomerization of Aβ42 but not Aβ40. The Aβ42 oligomer then causes caspase-3 activation. Propofol inhibits the isoflurane's effects on promoting Aβ42 oligomerization, leading to the attenuation of the isoflurane-induced caspase-3 activation.

Collectively, these results suggest that isoflurane may induce cytotoxicity via its effects on promoting Aβ42 oligomerization, which are consistent with the findings from other studies [17], [36], [37]. Moreover, these results suggest that propofol may attenuate the isoflurane-induced caspase-3 activation by diminishing the isoflurane-induced Aβ42 oligomerization (Figure 5).

## Discussion

The commonly used inhalation anesthetic isoflurane has previously been shown to induce caspase-3 activation and apoptosis [18], [19], [34], [36], [37], [38], [39]. The underlying mechanisms and interventions to attenuate such effects remain largely to be determined. Propofol, the most commonly used intravenous anesthetic, has been shown to protect against caspase activation and apoptosis [26], [27], [28], [29], [30], [31], [32]. We therefore investigated the effects of propofol on the isoflurane-induced caspase-3 activation *in vitro* and *in vivo*, since caspase-3 activation alone, without apoptosis, may also contribute to neuropathogenesis of AD dementia and cognitive dysfunction [14].

We have found that propofol can attenuate the isoflurane-induced caspase-3 activation. Other studies have shown that isoflurane impairs learning and memory in rodents [15], [16], [40], whereas propofol does not [41], [42]. Taken together, these findings suggest that propofol could be preferably used in patients who are susceptible to cognitive dysfunction, e.g., AD patients and older adults, pending further studies. Future research may also include assessing whether isoflurane alone can induce neurotoxicity and impairment of cognitive function, whereas isoflurane plus propofol will not, in both animal and human studies.

It is interesting that propofol can only attenuate the isoflurane-induced caspase-3 activation when levels of Aβ are already elevated (H4-APP cells and AD Tg mice). Other studies have shown that propofol can maximally attenuate the damage from cerebral ischemia, via its antioxidant effects, during the time of reperfusion when free radicals were markedly increased [31]. Thus, it is conceivable that propofol may execute its protective effects when the damage is high, e.g., elevated levels of Aβ and oxidative stress.

Elevated Aβ levels lead to more Aβ oligomerization and aggregation. Isoflurane has been shown to increase Aβ accumulation [18], [19], [37] and enhance Aβ oligomerization [17]. In addition, the inhibition of Aβ oligomerization and aggregation can attenuate the isoflurane-induced caspase-3 activation in cultured cells [36], [37]. Finally, in the current studies we have shown that propofol can attenuate the isoflurane-induced Aβ42 oligomerization. Collectively, we have postulated that one of the mechanisms by which isoflurane induces caspase-3 activation is to enhance Aβ oligomerization, especially when the Aβ levels are elevated. Propofol may attenuate this isoflurane-induced caspase-3 activation via its ability to reduce the isoflurane-induced Aβ oligomerization.

It has been reported, however, that high concentrations of propofol itself can increase Aβ42 oligomerization [17]. Therefore, it is possible that the effects of propofol on isoflurane-induced Aβ oligomerization are dose-dependent, and that high concentrations of propofol may potentiate, whereas low concentrations of propofol may attenuate, the isoflurane-induced Aβ oligomerization. Further studies are necessary to test these hypotheses, and to investigate the potential application of propofol-induced reduction in Aβ oligomerization.

It is also interesting that isoflurane only induced oligomerization of Aβ42, but not Aβ40, in the current studies. This observation may not be due to different concentrations of Aβ40 and Aβ42, because we found that there was no significant difference between the concentrations of Aβ40 and Aβ42 in the current studies. The exact underlying mechanisms of these findings remain to be determined. Previous studies by Yan and Wang [43] showed that the C terminus of Aβ42 is more rigid than the C terminus of Aβ40, and Aβ42 may have lower configurational entropy than Aβ40 in the C terminus under physiological conditions. Aβ42 would be easier to oligomerize than Aβ40 because the entropic price for Aβ42 to aggregate into fibrils is smaller than that for Aβ40. Therefore, it is conceivable that isoflurane may have more interaction with the C terminus of Aβ42 and less with Aβ40, owing to the greater rigidity of Aβ42 and weaker rigidity of Aβ40 C terminuses, which leads to more and less (or no) oligomerization of Aβ42 and Aβ40, respectively. Furthermore, propofol may interrupt the interaction of isoflurane with the C terminus of Aβ42, leading to attenuation of the isoflurane-induced Aβ42 oligomerization. More studies are needed to further test this hypothesis.

Propofol has been reported to have neuroprotective effects through activating GABA receptors [27] or by decreasing glutamate extracellular concentrations [32]. It has been further suggested that propofol may affect the anti-apoptotic signaling proteins, including Bcl-2, pAkt, and pERK, to have neuroprotective effects [29]. Isoflurane has been suggested to induce caspase-3 activation through other mechanisms, e.g., mitochondrial damages. Therefore, it is also possible that propofol can attenuate the isoflurane-induced caspase-3 activation via different mechanisms other than reducing Aβ42 oligomerization.

There are several limitations to the current studies. First, in the *in vivo* studies, we only assessed the effects of propofol on the isoflurane-induced caspase-3 activation in neonatal WT and AD Tg mice, rather than the adult WT and AD Tg mice. This is because the neonatal AD Tg mice are less expensive and easier to obtain. Furthermore, we have demonstrated that another inhalation anesthetic sevoflurane can induce a greater degree of caspase-3 activation in the AD Tg neonatal mice than in WT neonatal mice [44]. Second, we did not use several doses of propofol in the experiments. Rather, we used one dose of propofol to prove the concept that propofol can attenuate isoflurane-induced caspase-3 activation. The goal of the animal studies in the manuscript was to determine the *in vivo* relevance of the *in vitro* findings that propofol may attenuate isoflurane-induced caspase-3 activation in cells with elevated Aβ levels (H4-APP cells). The dose of propofol (200 mg/kg) in animals was equivalent to the dose of propofol (100 nM) in cultured cells and we found that both the *in vitro* and *in vivo* doses of propofol were able to attenuate isoflurane-induced caspase-3 activation. Nevertheless, this single dose of propofol used in the current experiment has allowed us to demonstrate that propofol may attenuate isoflurane-induced caspase-3 activation in the presence of elevated Aβ levels. In the future studies, we will systemically assess the effects of anesthetics, including propofol, on AD neuropathogenesis in both WT and AD Tg mice.

### Conclusion

We have found that the commonly used intravenous anesthetic propofol can attenuate the isoflurane-induced caspase-3 activation *in vitro* and *in vivo*. Moreover, propofol attenuates the isoflurane-induced caspase-3 activation only in the condition of elevated Aβ levels. Finally, propofol may mitigate the isoflurane-induced Aβ42 oligomerization. Collectively, these results suggest that isoflurane may induce caspase-3 activation via its ability to enhance Aβ42 oligomerization and that propofol can attenuate the isoflurane-induced caspase-3 activation through its ability to mitigate the isoflurane-induced Aβ42 oligomerization. These findings may lead to more studies aiming at the investigation of the underlying mechanisms and interventions of potential anesthesia neurotoxicity, which will ultimately provide safer anesthesia care and better postoperative outcome to patients.

## Materials and Methods

### H4 naïve and H4-APP cells

We employed H4 human neuroglioma cells (H4 naïve cells, purchased from American Type Culture Collection, Manassas, VA) and H4 naïve cells stably-transfected to express full-length (FL) human amyloid precursor protein (APP) (H4-APP cells) in the experiments. The H4 naïve cells were cultured in Dulbecco's Modified Eagle Medium (high glucose) containing 9% heat-inactivated fetal calf serum, 100 units/ml penicillin, 100 µg/ml streptomycin, and 2 mM L-glutamine. The H4-APP cells were cultured in the same cell culture supplemented with 220 µg/ml G418.

### Treatments for the H4 naïve and H4-APP cells

H4 naïve or H4-APP cells were placed in six well plates with 0.5 million cells and 1.5 ml cell culture media in each well. The cells were treated with a clinically relevant concentration of propofol (Diprivan, AstraZeneca Pharmaceuticals, Wilmington, DE), (100 nM) [32] with or without 2% isoflurane for six hours (air component: 2% isoflurane, 5% CO_2_, 21% O_2_ and balanced Nitrogen) as described by Xie et al. [19]. Isoflurane was delivered from an anesthesia machine to a sealed plastic box in a 37°C incubator. A Datex infrared gas analyzer (Puritan-Bennett, Tewksbury, MA) was used to continuously monitor the delivered concentrations of carbon dioxide, oxygen, and isoflurane. Propofol was prepared in 10% intralipid in DMEM and was administrated to cells 10 minutes before treatment with isoflurane.

### Cell lysis and protein amount quantification

The pellets of the harvested H4 naïve and H4-APP cells were detergent-extracted on ice using an immunoprecipitation buffer (10 mM Tris-HCl, pH 7.4, 150 mM NaCl, 2 mM EDTA, 0.5% Nonidet P-40) plus protease inhibitors (1 µg/ml aprotinin, 1 µg/ml leupeptin, 1 µg/ml pepstatin A). The lysates were collected, centrifuged at 13,000 rpm for 15 min, and quantified for total proteins by a bicinchoninic acid protein assay kit (Pierce, Iselin, NJ).

### Mice anesthesia

The animal protocol was approved by Massachusetts General Hospital Standing Committee (Boston, Massachusetts) on the Use of Animals in Research and Teaching (Approval ID: 2006N000219). The anesthesia used on the neonatal mice was similar to that described by Lu et al. [44]. Specifically, wild-type (WT) mice [C57BL/6J mice (The Jackson Laboratory, Bar Harbor, ME)] and AD transgenic (Tg) mice [B6.Cg-Tg(APPswe, PSEN1dE9)85Dbo/J, (The Jackson Laboratory, Bar Harbor, ME)] were distinguished by genotyping. All animals (3 to 6 mice per experiment) were six days old at the time of anesthesia and were randomized by weight and gender into experimental groups that received 1.4% isoflurane in 100% O_2_ [18] for six hours in an anesthetizing chamber or a control group that received 100% O_2_ [18] for six hours in a similar chamber with identical flow rate. Saline or propofol [(200 mg/kg, intraparietal injection (i.p.)] was administered to the mice 10 minutes before the isoflurane treatment, similarly to the methods described in other studies [45]. The mice breathed spontaneously, and anesthetic and O_2_ concentrations were measured continuously (Datex). The temperature of the anesthetizing chamber was controlled to maintain rectal temperature of the animals at 37±0.5°C. Mice were sacrificed by decapitation immediately after the isoflurane anesthesia. The brain was removed rapidly and the prefrontal cortex was dissected out and frozen in liquid nitrogen for subsequent processing for the determination of caspase-3 protein levels.

### Brain tissue lysis and protein amount quantification

The harvested brain tissues were homogenized on ice using an immunoprecipitation buffer (10 mM Tris-HCl, pH 7.4, 150 mM NaCl, 2 mM EDTA, 0.5% Nonidet P-40) plus protease inhibitors (1 µg/ml aprotinin, 1 µg/ml leupeptin, 1 µg/ml pepstatin A). The lysates were collected, centrifuged at 12,000 rpm for 10 min, and quantified for total proteins using a bicinchoninic acid protein assay kit (Pierce, Iselin, NJ).

### Western blots analysis

The harvested H4 naïve, H4-APP cells, and brain tissues were subjected to Western blot analyses as described by Xie et al. [46]. A caspase-3 antibody (1∶1,000 dilution; Cell Signaling Technology, Inc.) was used to recognize FL-caspase-3 (35–40 kDa) and caspase-3 fragment (17–20 kDa) resulting from cleavage at asparate position 175. Caspase-3 activation is defined as the quantitative ratio of caspase-3 fragment to caspase-3 full length, as only this ratio accurately represents the inter-conversion of full length caspase-3 to cleaved caspase-3, and therefore indicating caspase-3 activation. The antibody anti-β-Actin (1∶10,000, Sigma, St. Louis, MO) was used to detect β-Actin (42 kDa). Each band in the Western blot represents an independent experiment. We have averaged results from three to six independent experiments. The quantification of Western blots was performed as described by Xie et al. [19]. Briefly, the intensity of the signals was analyzed using the National Institute of Health image program (National Institute of Health Image 1.62, Bethesda, MD). We quantified the Western blots using two steps. First, we used levels of β-Actin to normalize (e.g., determining ratio of FL-caspase-3 amount to β-Actin amount) levels of caspase-3 to control for any loading differences in total protein amounts. Second, we presented changes in the levels of caspase-3 in treated cells or mice as folds of those in cells or mice in the control condition.

### The Aβ oligomerization assessment

The H4 Aβ40 overexpressed cells and H4 Aβ42 overexpressed cells [47] were placed in different 150 mm cell culture dishes with 10 ml cell culture media. We collected the media 48 hours later, when the confluence of the cells was about 75%. 1.5 ml of the media was placed in each well of a six well plate. The media were treated with a clinically relevant concentration of propofol (100 nM) [32] and/or 2% isoflurane for six hours. Then, we took a sample of 75 µl of the conditioned media from each well and added 25 µl loading buffer [4×sodium dodecyl sulfate polyacrylamide gel electrophoresis (SDS)+8% beta-mercaptoethanol (BME)] into the sample. Finally, 10 µl of the mixture of the sample and loading buffer was loaded and resolved in each lane on SDS- sodium dodecyl sulfate polyacrylamide gel electrophoresis (PAGE), transferred to polyvinylidene difluoride membrane (Bio-Rad Laboratories, Hercules, CA, U.S.A.), fixed with glutaraldehyde (1%, vol/vol), probed with the anti-Aβ monoclonal antibody (mAb) 6E10 (1∶250) (Covance, Dedham, MA, U.S.A.), and analyzed for SDS-stable oligomers according to the method of Atwood *et al.* [48].

### Statistics

Given the presence of background caspase activation in the cells, we did not use absolute values to describe changes in caspase activation. Instead, caspase-3 activation (ratio of caspase-3 fragment to caspase-3 full length) was presented as a fold of those of the control group. One fold of ratio of caspase-3 fragment to caspase-3 full length refers to control levels for the purposes of comparison to the experimental conditions. We presented changes in levels of caspase activation in the treated cells or mice as a fold of those in the cells or mice after the control condition. Data were expressed as the mean ± the standard deviation. The number of samples varied from three to six, and the samples were normally distributed. Two-tailed t-test and two-way ANOVA with post-hoc (Tukey) test [SAS software (Cary, NC, USA)] were used to compare the differences between groups. P values less than 0.05 (* or #) and 0.01(** or ##) were considered statistically significant.

## Supporting Information

Figure S1 **Comparison of the effects of propofol on the isoflurane-induced caspase-3 activation in the H4-APP cells and H4 naïve cells.** *A.* In H4 naïve cells (lanes 1 and 2), the propofol treatment (lane 2) does not attenuate the isoflurane-induced caspase-3 activation (lane 1). In H4-APP cells (lanes 3 and 4), the propofol treatment (lane 4) attenuates the isoflurane-induced caspase-3 activation (lane 3). Isoflurane induces a greater degree of caspase-3 activation in the H4-APP cells as compared to that in H4 naïve cells (lanes 1 versus lane 3). There is no significant difference in amounts of β-Actin in the isoflurane plus saline or isoflurane plus propofol-treated H4 naive or H4-APP cells. *B.* The quantification of the Western blot shows that propofol (gray bar) does not attenuate the isoflurane-induced caspase-3 activation (white bar) in the H4 naïve cells. However, in the H4-APP cells, isoflurane induces a greater degree of caspase-3 activation (black bar, * P = 0.042) as compared to that in H4 naïve cells (white bar), and two-way ANOVA shows that propofol attenuates the isoflurane-induced caspase-3 activation in the H4-APP cells (net bar, ## P = 0.004). (N = 4).
(TIF)

Figure S2 **Isoflurane induces caspase-3 activation in brain tissues of WT and AD transgenic (Tg) neonatal mice.** *A.* Isoflurane (lanes 3 to 5) induces caspase-3 activation as compared to control condition (lanes 1 and 2) in brain tissues of WT neonatal mice. There is no significant difference in amounts of β-Actin in the isoflurane or control condition treated-mice. *B.* The quantification of the Western blot shows that isoflurane (black bar, * P = 0.012) induces caspase-3 activation as compared to control condition in brain tissues of WT neonatal mice. (N = 4). *C.* Isoflurane (lanes 4 to 6) induces caspase-3 activation as compared to control condition (lanes 1 to 3) in brain tissues of AD Tg neonatal mice. There is no significant difference in amounts of β-Actin in the isoflurane or control condition treated-mice. *B.* The quantification of the Western blot shows that isoflurane (black bar, * P = 0.045) induces caspase-3 activation as compared to control condition in brain tissues of AD Tg neonatal mice. (N = 4).
(TIF)
