# Supplementary figures and images for: Anesthetic Propofol Attenuates the Isoflurane-Induced Caspase-3 Activation and Aβ Oligomerization
Source: PLoS One. 2011 Nov 1;6(11):e27019. doi: 10.1371/journal.pone.0027019 (PMC3206055; doi:10.1371/journal.pone.0027019)

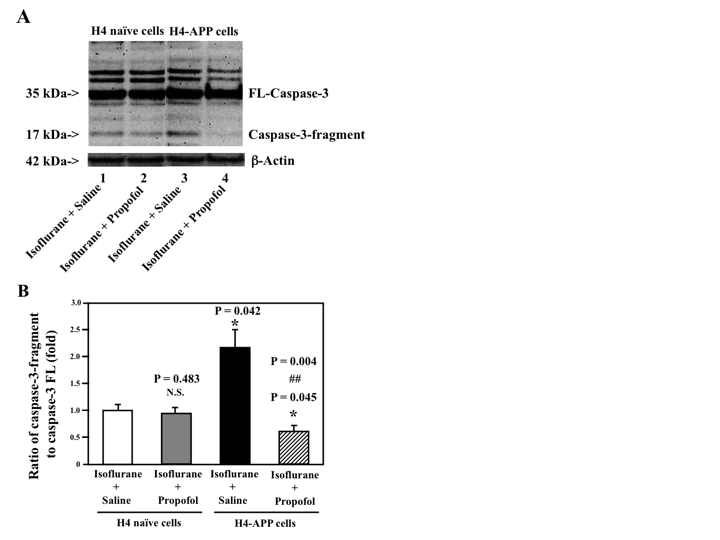

Supplement: Figure S1 — Comparison of the effects of propofol on the isoflurane-induced caspase-3 activation in the H4-APP cells and H4 naïve cells. A. In H4 naïve cells (lanes 1 and 2), the propofol treatment (lane 2) does not attenuate the isoflurane-induced caspase-3 activation (lane 1). In H4-APP cells (lanes 3 and 4), the propofol treatment (lane 4) attenuates the isoflurane-induced caspase-3 activation (lane 3). Isoflurane induces a greater degree of caspase-3 activation in the H4-APP cells as compared to that in H4 naïve cells (lanes 1 versus lane 3). There is no significant difference in amounts of β-Actin in the isoflurane plus saline or isoflurane plus propofol-treated H4 naive or H4-APP cells. B. The quantification of the Western blot shows that propofol (gray bar) does not attenuate the isoflurane-induced caspase-3 activation (white bar) in the H4 naïve cells. However, in the H4-APP cells, isoflurane induces a greater degree of caspase-3 activation (black bar, * P = 0.042) as compared to that in H4 naïve cells (white bar), and two-way ANOVA shows that propofol attenuates the isoflurane-induced caspase-3 activation in the H4-APP cells (net bar, ## P = 0.004). (N = 4). (TIF) [file pone.0027019.s001.tif]

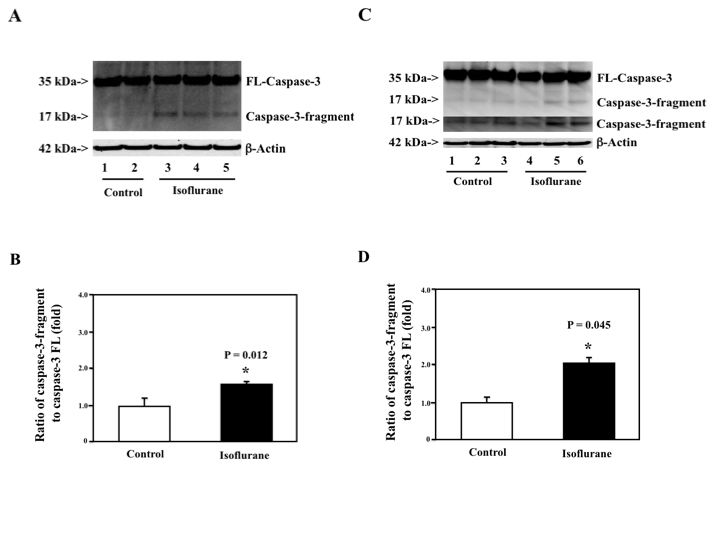

Supplement: Figure S2 — Isoflurane induces caspase-3 activation in brain tissues of WT and AD transgenic (Tg) neonatal mice. A. Isoflurane (lanes 3 to 5) induces caspase-3 activation as compared to control condition (lanes 1 and 2) in brain tissues of WT neonatal mice. There is no significant difference in amounts of β-Actin in the isoflurane or control condition treated-mice. B. The quantification of the Western blot shows that isoflurane (black bar, * P = 0.012) induces caspase-3 activation as compared to control condition in brain tissues of WT neonatal mice. (N = 4). C. Isoflurane (lanes 4 to 6) induces caspase-3 activation as compared to control condition (lanes 1 to 3) in brain tissues of AD Tg neonatal mice. There is no significant difference in amounts of β-Actin in the isoflurane or control condition treated-mice. B. The quantification of the Western blot shows that isoflurane (black bar, * P = 0.045) induces caspase-3 activation as compared to control condition in brain tissues of AD Tg neonatal mice. (N = 4). (TIF) [file pone.0027019.s002.tif]
